# Myeloid cell-derived LL-37 promotes lung cancer growth by activating Wnt/β-catenin signaling: Erratum

**DOI:** 10.7150/thno.82877

**Published:** 2023-06-21

**Authors:** Ping Ji, Yongxin Zhou, Yibao Yang, Junlu Wu, Hao Zhou, Wenqiang Quan, Junjun Sun, Yiwen Yao, Anquan Shang, Chenzheng Gu, Bingjie Zeng, Jenni Firrman, Weidong Xiao, Robert Bals, Zujun Sun, Dong Li

**Affiliations:** 1 Department of Clinical Laboratory, Shanghai Tongji Hospital, Tongji University School of Medicine, Shanghai 200065, China.; 2 Department of Thoracic-cardiovascular Surgery, Tongji hospital,Tongji University School of Medicine, Shanghai 200065, China.; 3 Department of Pharmacy, Putuo People's Hospital, Shanghai 200060, China.; 4 Dairy and Functional Foods Research Unit, Eastern Regional Research Center, Agriculture Research Service, United States Department of Agriculture, Wyndmoor, PA 19038, USA.; 5 Sol Sherry Thrombosis Research Center, Temple University, Philadelphia, PA 19140, USA.; 6 Department of Internal Medicine V-Pulmonology, Allergology, Respiratory Intensive Care Medicine, Saarland University Hospital, Homburg 66424, Germany.

We apologize that the original version of the above article contains errors that need to be corrected. The representative immunostaining images of Ki67 in the 10 ug LL-37 and 10ug LL-37 +XAV939 groups in Figure 6A were mis-pasted when choosing representative images from the image data. We confirm that these corrections made in this erratum do not affect the original conclusions. All the authors of the paper have agreed to this correction. The corrected Figure 6A appears below.

## Figures and Tables

**Figure 6:**
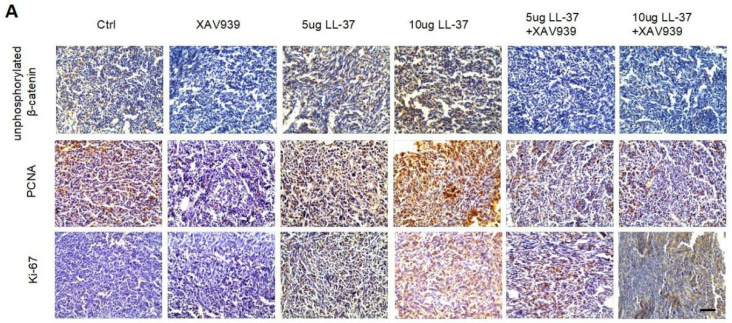
(A) Immunohistochemical analysis of the expression of unphosphorylated β-catenin, PCNA, and Ki67 in C57BL/6 mice inoculated with 2×105 LLC cells with indicated treatments. Scale bar, 50 μm.

